# Using quartz sand to enhance the removal efficiency of *M. aeruginosa* by inorganic coagulant and achieve satisfactory settling efficiency

**DOI:** 10.1038/s41598-017-14143-z

**Published:** 2017-10-19

**Authors:** Haiyan Pei, Yan Jin, Hangzhou Xu, Chunxia Ma, Jiongming Sun, Hongmin Li

**Affiliations:** 1 School of Environmental Science and Engineering, Shandong University, Jinan, 250100 China; 2 Shandong provincial engineering center on Environmental Science and Technology, Jinan, 250061 China

## Abstract

In this study, low-cost and non-polluting quartz sand was respectively mixed with AlCl_3_, FeCl_3_ and PAFC to synergistically remove *Microcystis aeruginosa*. Results showed that quartz sand could markedly increase the algae removal efficiency and decrease the coagulant doses. The increase of removal efficiency with AlCl_3_ and FeCl_3_ was only due to the enhancement of floc density by the quartz sand. However, the removal efficiency with PAFC was increased not only by the enhanced floc density, but also by the enlarged floc size. Flocs from 50 mg/L sand addition were larger than that with other sand doses, which was on account of the appropriate enhancement of collision efficiency at this dose. After coagulation, the extracellular organic matter (EOM) and microcystins (MCs) in system with quartz sand was remarkably reduced. That’s because quartz sand can enhance the coagulation so as to improve capping the EOM and MCs in flocs during coagulation process. Owing to 200 mg/L quartz sand could damage the cell’s membrane during coagulation proces, algal cells in the system lysed two days earlier than with 50 mg/L sand during flocs storage. In addition, cells with PAFC incurred relatively moderate cellular oxidative damage and could remain intact for longer time.

## Introduction

Over the past years, the presence of harmful algal blooms (HABs) in water has represented a big challenge to drinking water plants. These blooms always result in some serious issue, such as deterioration of water quality, off-flavor problems, and illness in animals and humans^1,2^. Among the harmful algae, the most notorious one is *M. aeruginosa*, which can produce a suite of hepatotoxins known as microcystins (MCs)^3^. MCs may cause serious health problems in humans through the consumption of contaminated water for drinking and recreation^4,5^.

In drinking water treatment plants, the coagulation process is an effective way to remove harmful algae^6^. However, due to the buoyancy of algal cells^7^, the flocs cannot be completely settled, which will reduce the removal efficiency. Recently, the application of ballast coagulation to control HABs has been extensively studied^8–10^. It is a gravity-based physicochemical separation process involves the injection of a ballasting agent, typically microsand, to increase the floc density and size^11^. For example, the combination of chitosan with local soils and clays achieved higher removal efficiencies of cyanobacteria in Taihu Lake^8–10^. The strong removal of harmful marine algae using chitosan mixed with sand has also been reported^12^. Compared to soils and clays, sands are more commercially viable and completely harmless to water quality. Ballast particles, such as clay, soil and sand, serve as seed grains when coagulation proceeds, while accelerating the sedimentation through increasing floc density^13^.

Until now, most researchers have focused on the use of ballast coagulation to remove harmful algae from natural water, rather than use in drinking water treatment. More issues need to be considered for application in drinking water treatment. For example, the ballast must not cause secondary pollution in drinking water. Therefore local soils are not suitable, because the metal ions and organic matter in soils may be released into drinking water. Besides, the ballast should not accelerate algal cell lysis and algal toxin release during floc storage, which would degrade the quality of the water obtained upon dewatering the flocs (*i.e*. the ‘dewatering water’).

Those researchers mainly combined organic coagulants (especially chitosan) with ballast to removal harmful algae. Although the removal efficiency was remarkable, the high-cost and increase of natural organic matter (NOM) would restrict the application of organic coagulants in drinking water treatment. Therefore, studying traditional inorganic coagulants combined with ballast to remove harmful algae is more meaningful in drinking water plant. AlCl_3_, FeCl_3_ and PAFC are the most widely used traditional organic coagulants in drinking water treatment. The cationic Al and Fe ion of these coagulants could coagulate negative charged algal cells through charge neutralization. Besides, as a polymeric coagulant, the bridging action of PAFC is also benefit for the coagulation. Hence, this study used low-cost and non-polluting quartz sand (also used in filter beds in drinking water plants) combined with AlCl_3_, FeCl_3_ or PAFC to remove *M. aeruginosa*.

The purposes of our research were to: (1) find the optimum coagulation conditions for AlCl_3_, FeCl_3_ and PAFC with different quartz sand doses; (2) verify the effects of coagulant type and quartz sand dose on floc properties; (3) reveal the effects of coagulants and quartz sand on dewatering water quality and cell integrity during floc storage.

In a nutshell, we strive to create a strategy called ‘sand plus inorganic coagulant’, which will not only reduce coagulant usage, but also form satisfactory flocs with good settling efficiencies and furthermore keep the algal cells intact for a longer time.

## Materials and Methods

### Materials

#### Cyanobacterial culture

*M. aeruginosa* FACHB-905 (purchased from Institute of Hydrobiology, Chinese Academy of Sciences (Wuhan, China)) was used as the model alga in this study. This strain was grown in BG11 medium at constant temperature (25 °C) with a light/dark cycle (12 h/12 h) under 2000 lux illumination. The cultures were harvested at the late exponential phase of growth and had a final cell yield of up to 10^7^ cells/mL (generally 10 days after inoculation). In this phase, cells have higher metabolic and enzymatic activity, are more consistent in size, and keep most MCs within the actively growing cells^14^.

#### Quartz sand

The quartz sand was purchased from Yan Feng Mineral Co. (Shijiazhuang, China). It was washed with deionized water, dried at 60 °C for 24 h, and then ground and sieved before use. The size distribution of quartz sand was that <38 μm: 85%, 38–75 μm: 9%, 75–150 μm: 6% (6%, 9% and 85% were mass percent). The density of quartz sand was around 2.66 g/cm^3^ and it was a nonconductor.

### Cyanobacterial coagulation

Coagulation experiments were performed with a six-paddle stirrer (ZR4-6, Zhongrun Water Industry Technology Development Co., China) in a series of 1000 mL beakers containing 500 mL of *M. aeruginosa* cultures. The initial *M. aeruginosa* concentration was diluted with deionized water to 4 × 10^5^ cells/L. The synthetic water quality parameters were: electrical conductivity 0.111 mS/cm, temperature 24.3 °C, pH 8.02, turbidity 6.64 NTU. After the coagulant (AlCl_3_, FeCl_3_ or PAFC) and quartz sand were added, the *M. aeruginosa* solution was stirred at 250 rpm for 1 min and 40 rpm for another 20 min. The doses of AlCl_3_ and PAFC used in the flocculation experiments were 1.0 to 3.5 mg/L and the doses of FeCl_3_ were 4.0 to 6.5 mg/L. The quartz sand doses in *M. aeruginosa* solution were 0, 20, 50, 100, and 200 mg/L. After sedimentation for 30 min, samples collected from 2 cm below the surface were characterised by OD_680_ using a UV-2450 spectrophotometer (Shimadzu, Japan) to indicate the removal efficiency:

$${\rm{Removal}}\,{\rm{efficiency}}=({{{\rm{OD}}}^{{\rm{a}}}}_{680}-{{{\rm{OD}}}^{{\rm{b}}}}_{680})/{{{\rm{OD}}}^{{\rm{a}}}}_{680}$$

The OD^a^_680_ was the OD_680_ of initial *M. aeruginosa* solution and the OD^b^_680_ was the OD_680_ of samples collected from 2 cm below the surface after coagulation and sedimentation.

The appropriate dose of each coagulant was then chosen to analyze the sedimentation properties of flocs with different quartz sand doses. After sedimentation for 0, 1, 3, 10, 30, 60, 120, 180 and 240 min, samples were collected from 2 cm below the surface to quantify the removal efficiency. All the coagulation experiments were conducted in triplicate and the results are presented as the mean values and standard deviations.

### Floc properties

A Mastersizer 2000 (Malvern, UK), was used to quantify dynamic floc size and size distribution during the coagulation process. Floc size measurements were taken every 30 s during the coagulation process and the results were recorded automatically by a computer. The size data were expressed as equivalent-volume diameters, and the floc sizes were characterized by the median equivalent-volume diameter (*d*_50_).

The floc formation, breakage and re-growth process can be divided into four regions: the rapid growth region, stable region, breakage region and restabilisation region^15^. The size distributions of flocs formed by AlCl_3_, FeCl_3_ and PAFC with different sand doses were measured at 18 min (in the stable region). The growth rate of floc in this study was calculated by the slope of the rapid growth region:

1 $${\rm{Growth}}\,{\rm{rate}}=\frac{{\rm{\Delta }}\mathrm{size}}{{\rm{\Delta }}\mathrm{time}}$$

The flocs’ strength and recoverability were indicated by a strength factor and recovery factor:

2 $${\rm{Strength}}\,{\rm{factor}}=\frac{{d}_{2}}{{d}_{1}}$$

3 $${\rm{Recovery}}\,{\rm{factor}}=\frac{{d}_{3}-{d}_{2}}{{d}_{1}-{d}_{2}}$$

where *d*_1_ is the average floc size in the stable region, *d*_2_ is the floc size after breakage, and *d*_3_ is the average floc size in the restabilisation region^16^.

The fractal dimension (*D*_f_) of aggregates was measured by the small-angle laser light scattering (SALLS) method in the Mastersizer 2000 with a 632.8 nm laser light beam^17^. The total scattered light intensity *I*, the scattering vector *Q*, and *D*_f_ are related by a power law^18^:

4 $$I\,{\rm{\alpha }}\,{{Q}^{D}}_{{\rm{f}}}$$

The scattering vector *Q* is the difference between the incident and scattered wave vectors of the radiation beam in the medium, which is given by the equation^19^:

5 $$Q=\frac{4\pi n\,\sin (\theta /2)}{\lambda }$$

where *n*, *θ* and *λ* are the refractive index of the medium, the laser light wavelength in a vacuum, and the scattering angle, respectively.

### Extracellular MCs

2 mL samples were centrifuged at 2300 *g* for 10 min to separate the dissolved MCs, and the concentrations of dissolved MCs in the centrates were measured using a Beacon Microcystin ELISA kit (Beacon Analytical Systems, Maine, USA)^20^. The detections of extracellular MCs were conducted in triplicate and the results are presented as the mean values and standard deviations.

### Extracellular organic matter (EOM)

The EOM was described by fluorescence excitation–emission matrices (EEM). 2 mL algal cell solution was sampled every second day and filtered through a 0.45 μm membrane (Xinya Co. Ltd, Shanghai, China) for EOM analysis with a fluorescence spectrophotometer (F-4600, Hitachi, Japan). The excitation wavelengths were increased from 200 to 450 nm in 5 nm steps, and the emission spectra were evaluated in 1 nm steps between 220 and 550 nm. Excitation and emission slits were both maintained at 5 nm and the scanning speed was set at 2400 nm/min^21^.

### Physiological indicators

During floc storage, 10 mL algal floc solution was sampled and centrifuged at 5900 *g* for 10 min to collect algal cells. Cells were then re-suspended with phosphate buffer (50 mM, pH 7.0) and homogenized by an ultrasonic cell pulverizer (JY92-2D, Xinzhi Co., China) under ice-bath cooling for a total time of 10 min (600 W; ultrasonic cycle time: 2 s; rest cycle time: 8 s). After that, the homogenate was centrifuged at 13400 *g* for 15 min and the cell-free enzyme supernatant was maintained at −20 °C for further use^22^. The intracellular protein was determined by the Bradford method^23^ using bovine serum albumin as standard. Malondialdehyde (MDA) content was measured using a colorimetric method^24^. Superoxide dismutase (SOD) activity was measured according to the method of Beauchamp and Fridovich^25^. Then, in each system, the MDA content and SOD activity respectively divided the intracellular protein to get the MDA content and SOD activity per unit protein^26^. The detections of MDA and SOD were conducted in triplicate and the results are presented as the mean values and standard deviations.

### Scanning electron microscopy

To directly assess the surface information and morphology of *M. aeruginosa* cells, a scanning electron microscope (SEM) was used. 4 mL sludge samples were centrifuged at 1500 *g* for 5 min, the pellets were pre-fixed with 2.5% glutaraldehyde overnight, washed by phosphate buffer solution three times and post-fixed with 1% osmium tetraoxide for 1 h, then washed again with phosphate buffer solution. After that, samples were consecutively treated with 50%, 75%, 90% and 100% ethanol solutions (15 min each) and dried with a vacuum drier. The completely dry samples were then mounted on a copper stub, coated with gold and examined with an SEM (S-4100, Hitachi, Japan) at 3 kV^26^.

## Results and Discussion

### Effects of coagulants and quartz sand on *M. aeruginosa* removal

**Figure 1:**
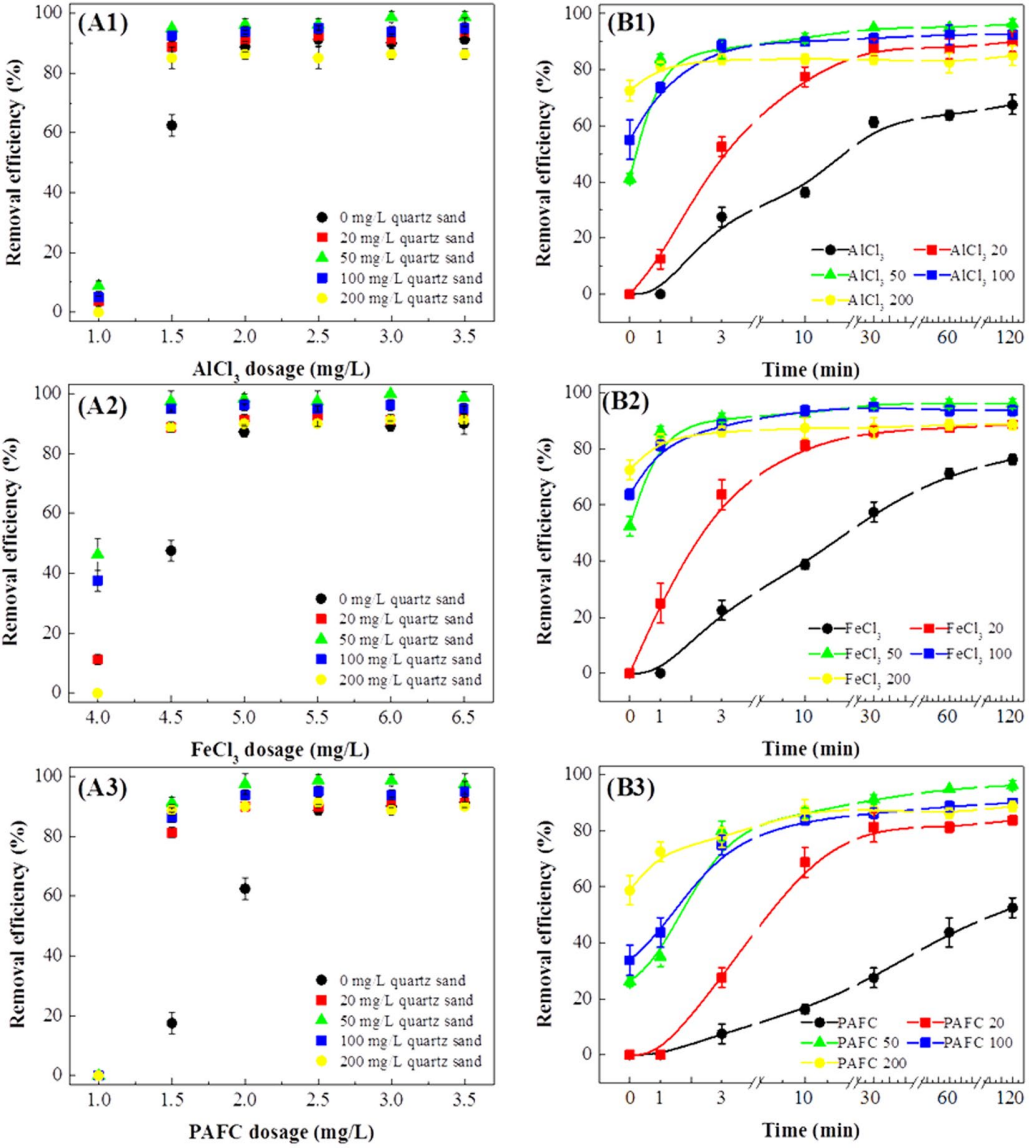
The removal efficiencies (**A**) and sedimentation kinetics (**B**) of algal cells for AlCl_3_ (A1, B1), FeCl_3_ (A2, B2) and PAFC (A3, B3) combined with different sand doses. (B1: “AlCl_3_” to “AlCl_3_ 200”, denoting 1.5 mg/L AlCl_3_ with 0 to 200 mg/L quartz sand. B2: “FeCl_3_” to “FeCl_3_ 200”, denoting 4.5 mg/L FeCl_3_ with 0 to 200 mg/L quartz sand. B3 “PAFC” to “PAFC 200”, denoting 1.5 mg/L PAFC with 0 to 200 mg/L quartz sand).

In Fig. 1A, it can be seen that the quartz sand could markedly increase *M. aeruginosa* removal efficiency, and coagulants with 50 mg/L sand doses achieved the best results. The optimum doses of coagulants with and without quartz sand were different: with quartz sand the optimum doses of AlCl_3_, FeCl_3_, and PAFC were 1.5 mg/L (0.3 mg/L as Al), 4.5 mg/L (1.55 mg/L as Fe), and 1.5 mg/L (0.24 mg/L as Al and 0.03 mg/L as Fe), while without quartz sand they were 2.0 mg/L (0.4 mg/L as Al), 5.0 mg/L (1.72 mg/L as Fe) and 2.5 mg/L (0.4 mg/L as Al and 0.05 mg/L as Fe), respectively. Therefore, with quartz sand the AlCl_3_, FeCl_3_ and PAFC doses could be reduced by 25%, 10% and 40%, respectively, and the quartz sand had the most marked effect on PAFC.

One of the reasons that quartz sand was able to improve the *M. aeruginosa* removal efficiency might be that mixing flocs with sand would enhance their sedimentation properties. Therefore, we chose the appropriate dose of each coagulant (AlCl_3_: 1.5 mg/L, FeCl_3_: 4.5 mg/L and PAFC: 1.5 mg/L) to assess the sedimentation behavior of flocs with different quartz sand doses. In Fig. 1B, the kinetic curves could directly reflect the different patterns of flocs sedimentation. It could be seen that without quartz sand, the flocs had a low and stable sedimentation velocity: 0.4% to 0.6% of total algal cells settled every minute (denoting 0.4 to 0.6%/min). Besides, their kinetic curves were more like straight lines, which mean the sedimentation kinetics curves could not reach a steady stage and the sedimentation could not be completed during 120 minutes. However, when adding quartz sand, the sedimentation velocities were remarkably increased and showed a rapid sedimentation stage at the beginning (9 to 42%/min). It might be because that the incorporation of sand to algal flocs would increase floc density so as to enhance the flocs sedimentation. After the rapid sedimentation, most algal cells had settled and the kinetic curves exhibit a steady stage. With 20 mg/L sand, the curves remain steady after 30 minutes sedimentation while with 50 to 200 mg/L, only 3 minutes were necessary. Thus, adding quartz sand would change the flocs sedimentation kinetics and significantly reduce the sedimentation time.

However, if quartz sand only affected the floc density, then a 200 mg/L quartz sand dose would have achieved the best *M. aeruginosa* removal efficiency, rather than 50 mg/L. Besides, the effect of quartz sand on AlCl_3_, FeCl_3_ and PAFC was obviously different. PAFC combined with quartz sand showed better removal efficiency than the other combinations. Therefore, we chose the appropriate dose of each coagulant to systematically analyze the floc properties for AlCl_3_, FeCl_3_ and PAFC coagulants with different quartz sand doses.

### Effects of coagulants and quartz sand on floc properties

**Figure 2:**
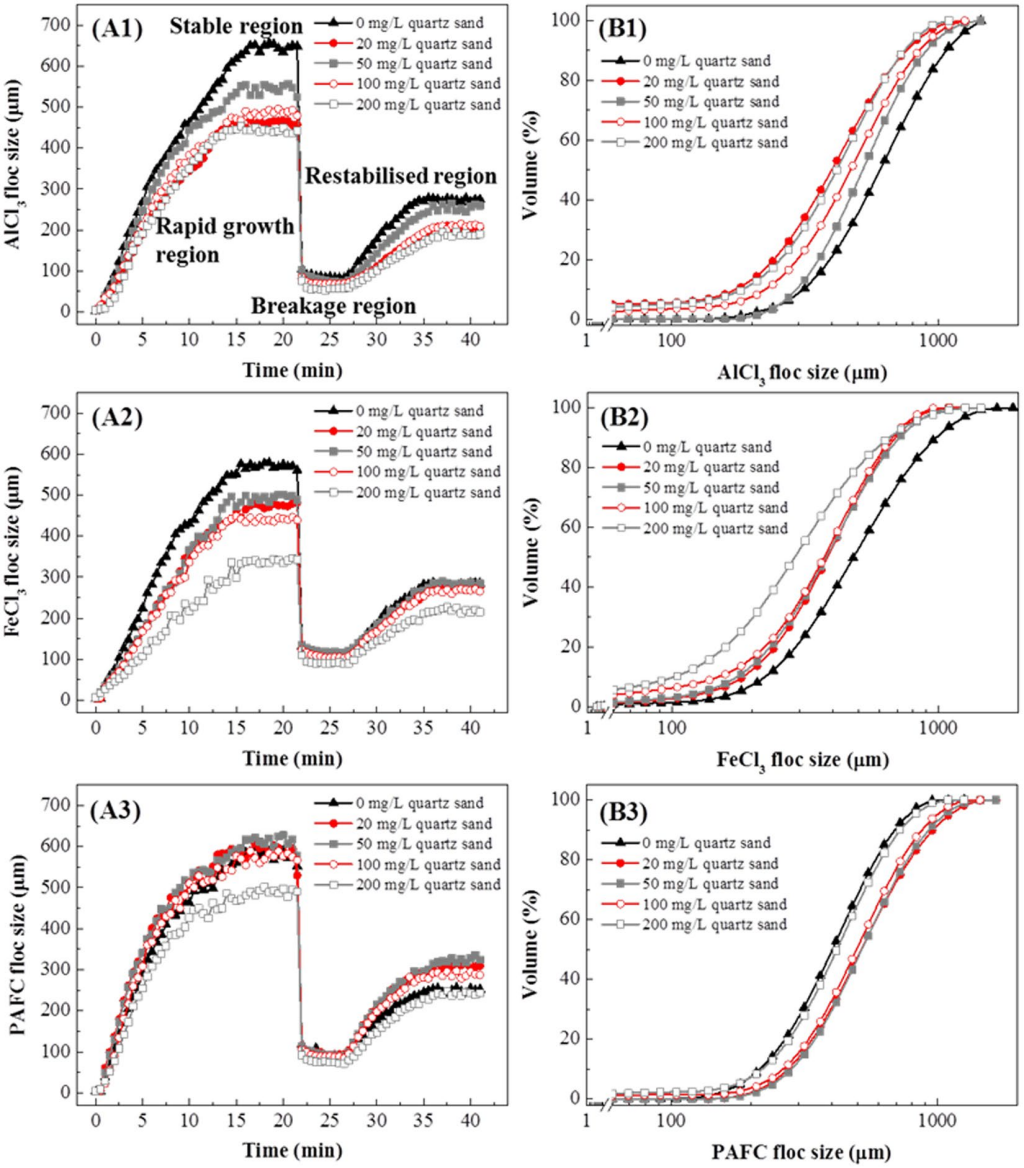
The floc size (**A1**–**A3**) and cumulative volume percent of floc size (**B1**–**B3**) with appropriate coagulant doses combined with different sand doses.

Figure 2A shows the size of flocs formed by AlCl_3_, FeCl_3_ and PAFC with different quartz sand doses, demonstrating the four regions described above. In coagulation processes, the floc size in the stable region is the most significant, as it decides the coagulation efficiency, because large flocs settle more easily and achieve better removal efficiencies^27^.

It can be seen that in the stable region, the sizes of flocs formed by AlCl_3_, FeCl_3_ and PAFC with 20 to 200 mg/L quartz sand are mostly consistent with the algae removal efficiencies. With 20 to 200 mg/L quartz sand, the floc sizes first rise and then fall as the sand dose increases; the largest floc size is formed at 50 mg/L sand, while the smallest floc size is formed at 200 mg/L sand. This explains why the algae removal efficiency would decrease with high sand doses: excessive quartz sand could decrease the flocs’ size, which makes it hard for them to settle.

Interestingly, comparing flocs with and without sand, the trends are different between AlCl_3_, FeCl_3_ and PAFC. The flocs formed by AlCl_3_ and FeCl_3_ at all of the sand doses are smaller than flocs without sand. Despite having a larger size, the flocs without sand also had a poor removal efficiency, because those flocs may float in the water column due to their buoyancy^7^, which the quartz sand can overcome. However, the size of flocs formed by PAFC with sand (except 200 mg/L) is larger than for flocs without sand. This explains why PAFC combined with quartz sand showed better removal efficiency than other coagulants: the sand not only increased the floc density but also enlarged the floc size.

W. Shi *et al*. reported that, as ballast, soil particles could enhance the collision frequency between particles, which is crucial to floc size^28,29^. Our research adds more details to this conclusion. Firstly, the ballast can only enlarge flocs formed by polymeric coagulants (PAFC). Secondly, only the appropriate ballast dose will increase the flocs’ size. Low doses of ballast had negligible effect on coagulation dynamics, while the excessively intense collision frequency with high doses of ballast will inhibit the formation of flocs.

The cumulative volume percentages for floc size in the stable region (at 18 min) allowed further verification of the conclusions above (Fig. 2B). For AlCl_3_ and FeCl_3_, the quartz sand (except 50 mg/L) would induce the production of small flocs, meaning less than 100 µm. These small flocs were hard to settle and reduced the algal cell removal efficiency. For PAFC, however, none of the doses of quartz sand would induce the production of small flocs. That is why PAFC combined with quartz sand showed better removal efficiency than the other combinations. Besides, 50 mg/L quartz sand did not induce the production of small flocs for any of the three coagulants, which would improve the algae removal efficiency.

**Figure 3:**
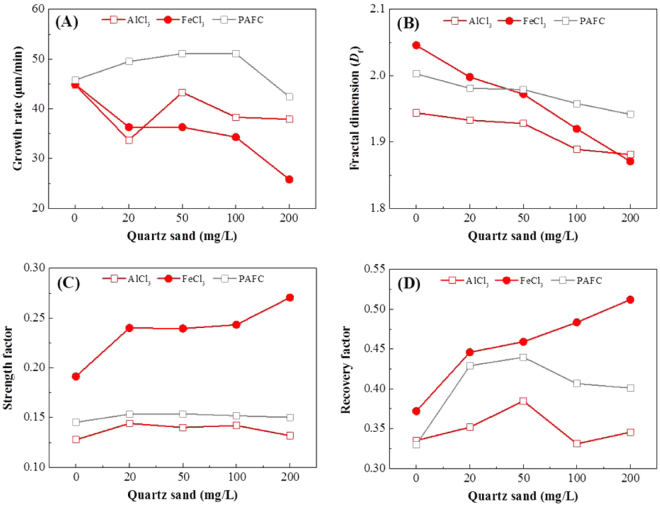
The growth rate (**A**), fractal dimension (**B**), strength factor (**C**) and recovery factor (**D**) of algal flocs with appropriate coagulant doses combined with different sand doses.

The floc growth rate, fractal dimension (*D*_f_), strength and recovery factor were calculated from floc size in the four regions (Fig. 3). Figure 3A shows the growth rate of flocs formed by AlCl_3_, FeCl_3_ and PAFC with different sand doses. Duan *et al*. reported that increasing the chance of collision and capturing other particles would lead to faster aggregation^30^. However, our research is contradictory on this point: the enhanced collision rate (as quartz sand dose increases) will not always increase the floc growth rate, which is decided by coagulant type and ballast dose.

For example, the growth rate of FeCl_3_ flocs decreases with increasing quartz sand dose, which indicates that the enhanced collision rate can conversely inhibit FeCl_3_ floc formation. The quartz sand can also decrease the growth rate of AlCl_3_ flocs, but the growth rate first rises and then drops for sand doses from 20 to 200 mg/L. In this range, flocs from 50 mg/L sand doses achieve the highest growth rate, but it is also slightly lower than for AlCl_3_ flocs without sand. Interestingly, quartz sand can increase the growth rate of PAFC flocs for sand doses from 0 to 100 mg/L, but excessive sand (200 mg/L) will also inhibit the formation of PAFC flocs. Therefore, only with polymeric coagulants and an appropriate ballast dose can the quartz sand increase the floc growth rate.

Figure 3B shows the fractal dimension (*D*_f_) of flocs in the stable region, which has a positive correlation with the flocs’ compactness. It can be seen that without quartz sand, FeCl_3_ flocs are more compact than AlCl_3_ and PAFC flocs, which is in accordance with the research of Gonzalez-Torres *et al*.^31^. However, when the quartz sand dose increases from 0 to 200 mg/L, the flocs’ compactness will accordingly decrease, and the FeCl_3_ flocs’ compactness drops more sharply than the others.

**Figure 4:**
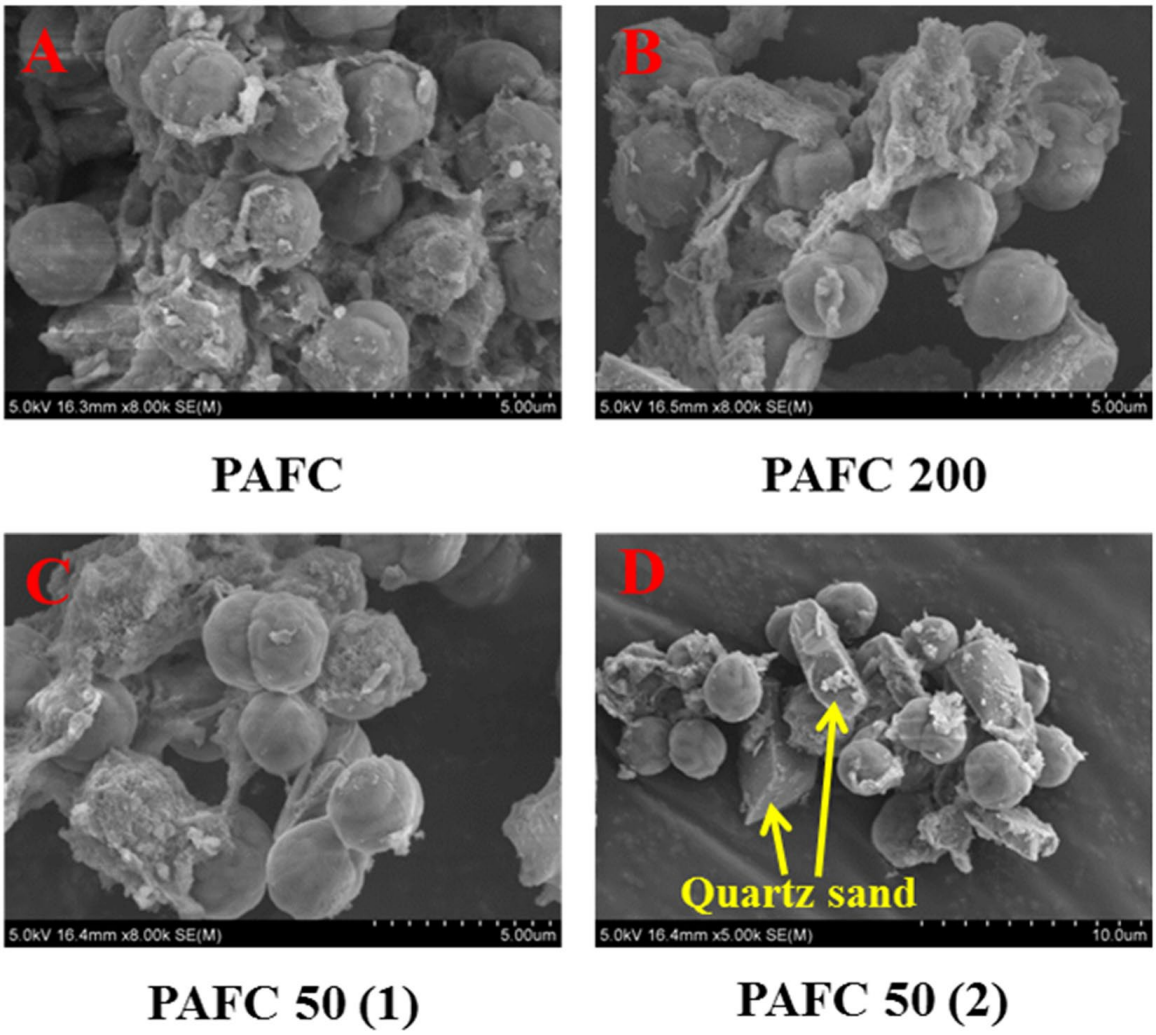
SEM micrographs of *M. aeruginosa* cells in coagulation process. (**A)** 1.5 mg/L PAFC only. (**B**) 1.5 mg/L PAFC with 200 mg/L quartz sand. (**C**,**D**) 1.5 mg/L PAFC with 50 mg/L quartz sand).

Until now, no research had reported results showing that ballast could reduce flocs’ compactness. Hence, from the SEM micrographs of *M. aeruginosa* flocs (Fig. 4), we surmise that the shape and size of particles forming flocs might affect the compactness of flocs. Without quartz sand, most of the particles are *M. aeruginosa* cells at the late exponential phase of growth. These algae cells are subspheroidal and have similar sizes. The similar shape and size make the particles arrange themselves in an orderly fashion within the flocs, which reduces the space between particles so as to achieve a more compact structure (Fig. 4A). However, the shape and size of quartz sand particles are irregular. When mixed with algae cells, the particles within flocs would be in a disorderly arrangement, and make the flocs less compact (Fig. 4B–D).

The strength factor reflects the ability of flocs to resist breakage under high shear rates, and the recovery factor shows the capacity to regrow to their original size under low shear rates after floc breakage (Fig. 3C and D). In the research of Gonzalez-Torres *et al*.^31^, when applying ferric as the coagulant, the floc strength and regrowth potential were far greater than for other coagulants, which was similar to our results without quartz sand.

When adding quartz sand, the change of strength and recovery factors for FeCl_3_ flocs was different from the behavior for AlCl_3_ and PAFC flocs. It can be seen that the strength and recovery factors of FeCl_3_ flocs consistently increase with increases in sand dose. However, the recovery factors of AlCl_3_ and PAFC flocs exhibit first a rise and then a fall for quartz sand doses from 0 to 200 mg/L, and the strength factors are not evidently changed. This indicates that appropriate sand dose would increase the regrowth potential of AlCl_3_ and PAFC flocs, while the strength of flocs were not depend on sand dose. Yet for FeCl_3_ flocs, quartz sand consistently increased the strength and regrowth potential for doses from 0 to 200 mg/L. It had been surmised that broken flocs were not generally able to reform to their original size because they tended to lose bonding capacity, resulting in fewer active bonding sites being available for reattachment^32^. Therefore, the greater strength and regrowth potential of FeCl_3_ flocs might be due to the more active bonding sites in FeCl_3_ flocs, which made algal cells easy to attach to quartz sand and hard to separate.

### Effects of coagulants and quartz sand on *M. aeruginosa* cells during floc storage

Studying the synergistic effects of coagulants and ballast on algal cells during floc storage is also important in drinking water treatment. If the Fe ions, Al ions or ballast could affect the lysis of *M. aeruginosa* cells, the increased EOM and extracellular MCs would pollute the supernatant recovered upon dewatering. In addition, the flocs formed by different coagulants and ballast doses have diverse protective effects. Therefore, in this part of the study we chose three coagulants with 0, 50, and 200 mg/L quartz sand doses and raw *M. aeruginosa* suspension to study the fate of algal cells in these samples during floc storage.

**Figure 5:**
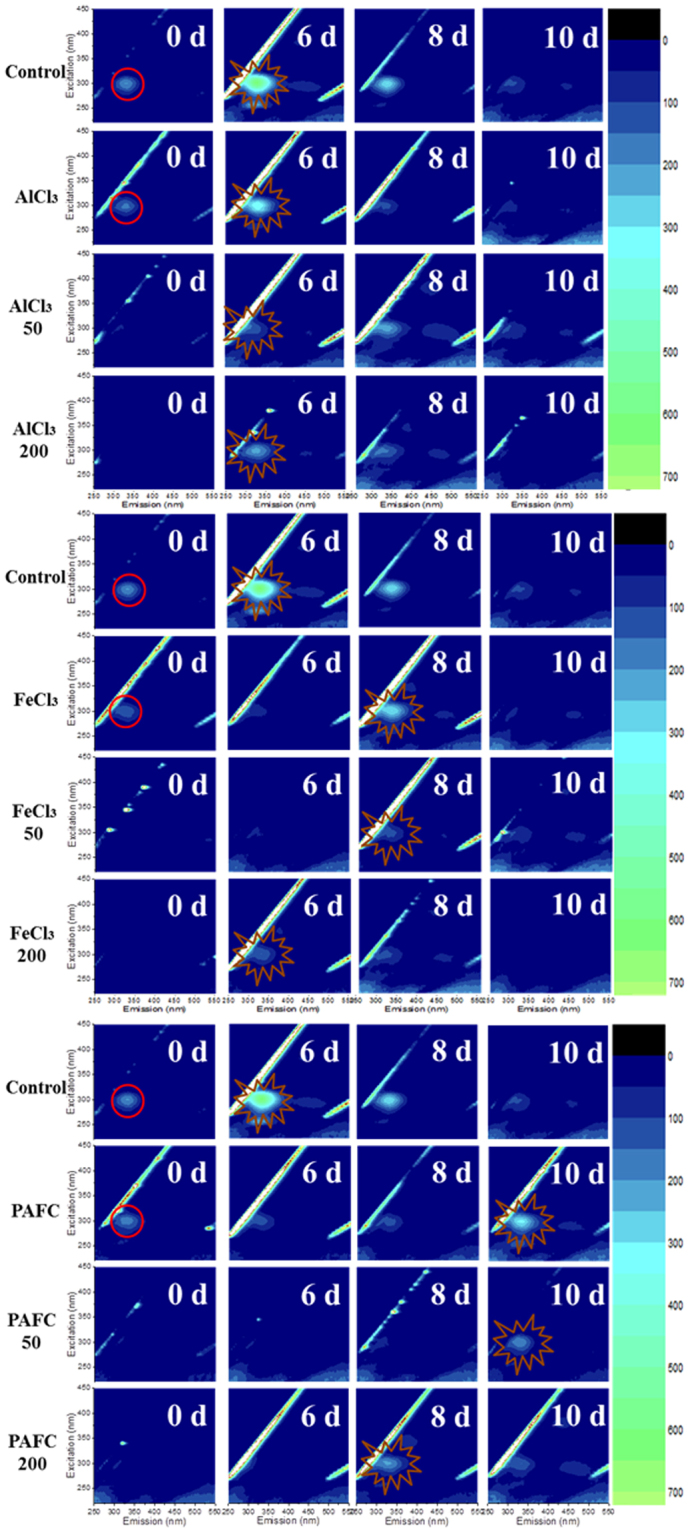
The EEM spectra of AlCl_3_, FeCl_3_ and PAFC flocs with 0, 50 and 200 mg/L quartz sand during 10 days’ storage.

Typical EEM fluorescence spectra imply the EOM of samples during floc storage. In our research, the major peak is observed for an Ex/Em of 270-280/305-310 nm in the EEM, which belongs to protein-like substances^33^ (Fig. 5). It can be seen that at 0 d (immediately after coagulation), there is little EOM content in samples with quartz sand, while an obvious protein peak is observed in samples without sand (marked with red circles). The reason was that quartz sand could enhance the coagulation so as to improve capping the EOM in flocs during coagulation process^34^. Thus, the samples with quartz sand had lower EOM contents during the 10 days’ storage, which would increase the quality of water recovered from dewatering the flocs. However, without sand this phenomenon was remarkably weakened^34^.

When most *M. aeruginosa* cells lysed, the intracellular organic matter would be largely released and make the EOM content reach maximum. Thus, the day EOM reach maximum could be considered as the day algal cells lysed. In Fig. 5, the lysis time of algal cells in samples without quartz sand is 6 d (AlCl_3_), 8 d (FeCl_3_) and 10 d (PAFC), respectively (marked with brown starbursts), which are similar to Xu’s observations. The variation in lysis times was because excessive Al and Fe were harmful to *M. aeruginosa* cells, but the optimal PAFC dose has benign levels of Al and Fe and the protective effect of PAFC flocs is better than that of AlCl_3_ and FeCl_3_ flocs^26^. However, when adding quartz sand, the cell lysis times also depended on the sand dose. For FeCl_3_ and PAFC flocs, the lysis times of cells with 50 mg/L sand dosages were the same as for cells without any sand dose, while the cells with 200 mg/L sand dosages lysed two days earlier. The reason might be that excessive concentrations of quartz sand (200 mg/L) would enhance the collision efficiency between algal cells and sharp quartz sand particles while stirring, which could damage the cell membranes.

SEM micrographs of *M. aeruginosa* cells taken from the coagulation process allow verification of the deduction above (Fig. S1). In Fig. S1, cells were just mixed with different doses of quartz sand. It can be seen that with 200 mg/L sand, some algal cells were destroyed by the collision with sharp quartz sand particles, while with 50 mg/L sand the cells were intact and healthy. Therefore, excessive concentrations of quartz sand (200 mg/L) would damage the cell’s membrane during coagulation process, thus make it lysing earlier during floc storage. However, the damage to cells at the appropriate sand dose (50 mg/L) was negligible. For AlCl_3_, cells with 50 mg/L and 200 mg/L doses of sand both lysed at 6 d, which was because compared to the toxic effect of Al, the harm of excessive quartz sand to cells was negligible.

**Figure 6:**
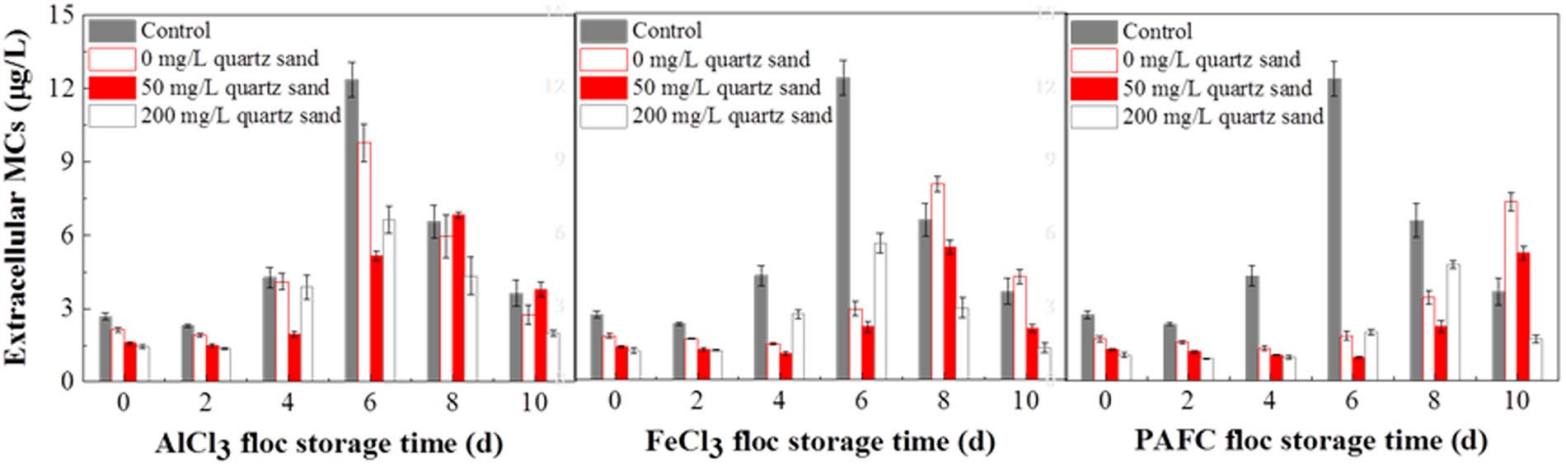
The extracellular MCs concentrations for AlCl_3_, FeCl_3_ and PAFC flocs with 0, 50, and 200 mg/L quartz sand during storage for 10 days (Control is algal solution without coagulants and quartz sand).

When treating raw water containing *M. aeruginosa*, it is critical to remove MCs^35^. In Fig. 6, the change of MCs during floc storage is similar to the trends observed for EOM. Because quartz sand could enhance the coagulation and improve capping the MCs in flocs during coagulation process^34^, the MCs content of samples with sand is lower than other samples, which will improve the water quality. But excessive sand doses (200 mg/L) will make cells lyse earlier and degrade the water quality. Besides this, cells coagulated with PAFC could remain intact for a longer time during floc storage. Therefore, it can be seen that application of PAFC combined with 50 mg/L quartz sand is a safer and more efficient way to perform ballast coagulation.

As we know, the cell membranes of algae are vulnerable to reactive oxygen species (ROS) in an adverse environment. As the coagulants and quartz sand could have an environmental stress on the algal cells, they would induce the algal cellular oxidative damage. Therefore, the extent of damage to algal cells from different coagulants with quartz sand can be further evaluated by assessing the cellular oxidative damage^22^. As the decomposition product of this process, MDA has been utilized frequently as a biomarker for cellular oxidative damage^36^: the MDA content has a positive correlation with the extent of cell damage. In Fig. S2(A), during floc storage, cells with PAFC have relatively moderate oxidative damage, while the damage to cells with AlCl_3_ is most serious. This is due to the toxic effect of Al and the excellent protective effect of PAFC flocs^26^. When adding quartz sand, the cellular oxidative damage becomes serious with increased sand dose, especially with 200 mg/L. In addition, without the protection of flocs, the cellular oxidative damage of raw algae solution is also serious.

Superoxide radicals and hydrogen can be used by SOD to produce hydrogen peroxide and oxygen, which has been described as the first line of resistance against ROS^37,38^. As shown in Fig. S2(B), SOD activity was induced by the coagulants and quartz sand in the initial stage in response to the stress. However, with extended time the antioxidant defense system was damaged and then the SOD activity deceased. It can be inferred that the SOD activity decreased earlier with more serious cellular oxidative damage. To sum up, the harm of quartz sand to algal cells in coagulation process could show during storage period, thus its dose should be controlled in ballast coagulation.

**Figure 7:**
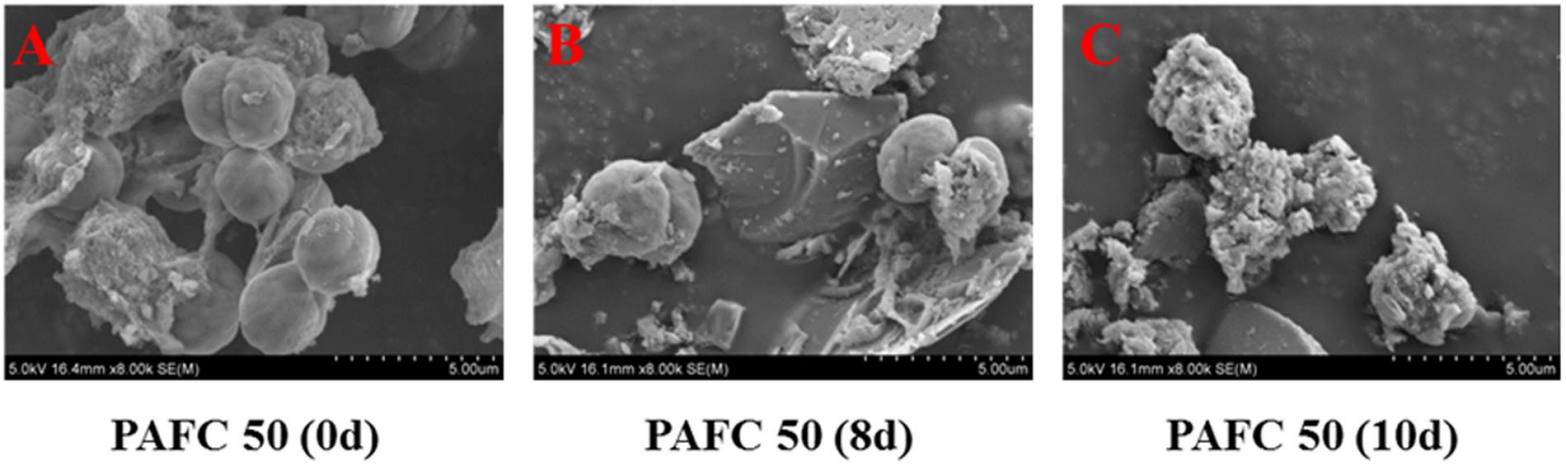
SEM micrographs of *M. aeruginosa* cells coagulated with 1.5 mg/L PAFC and 50 mg/L quartz sand stacked for 0 d (**A**), 8 d (**B**) and 10 d (**C**).

After coagulation with 1.5 mg/L PAFC and 50 mg/L quartz sand, the cells were stored for (A) 0 d, (B) 8 d, and (C) 10 d. The samples in the above systems were then taken for SEM analysis to assess the morphology changes in *M. aeruginosa* cells during floc storage (Fig. 7). It can be seen that on the 8^th^ day the algae cells still did not lyse. But the shape of cells was distorted and some of the cells were deflocculated. After 10 days, all of the cells were deflocculated and completely lysed. Thus, the *M. aeruginosa* cells would lyse with flocs storing and the reasons could be attributed to two sides: one was the loss of flocs protection due to the deflocculation of algal cells; the other one was that nutrient would be exhausted with storage, which made algal cells vulnerable and distorted cells’ shape. The conclusion coincides with the results from our previous study^14^.

## Conclusion

The low-cost and non-polluting quartz sand was used to increase the algae removal efficiency and reduce coagulant doses. With 1.5 mg/L PAFC and 50 mg/L quartz sand, the properties of algal flocs were better than in other conditions. During floc storage, with PAFC coagulant and 50 mg/L quartz sand, EOM and MC levels were obviously decreased, and the *M. aeruginosa* cells in flocs could remain intact for a longer time. Therefore, in this study PAFC with 50 mg/L quartz sand was the optimum condition, not only in the coagulation process but also for floc storage, and it might be a feasible treatment to implement in drinking water production.

## Electronic supplementary material

Supplementary Information
