# Supplementary material for: Using quartz sand to enhance the removal efficiency of M. aeruginosa by inorganic coagulant and achieve satisfactory settling efficiency
Source: Sci Rep. 2017 Oct 19;7:13586. doi: 10.1038/s41598-017-14143-z (PMC5648817; doi:10.1038/s41598-017-14143-z)
Supplement: Supplementary file 1 — Supplementary Information [file 41598_2017_14143_MOESM1_ESM.pdf]

Supplementary Information

Using quartz sand to enhance the removal efficiency of *M. aeruginosa* by inorganic coagulant and achieve satisfactory settling efficiency

Haiyan Pei[a, b] *, Yan Jin[a], Hangzhou Xu[a], Chunxia Ma[a], Jiongming Sun[a], Hongmin Li[a]

[a] *School of Environmental Science and Engineering, Shandong University, Jinan, 250100, China.*

[b] *Shandong provincial engineering center on Environmental Science and Technology, Jinan, 250061, China.*

* Corresponding author: School of Environmental Science and Engineering, Shandong University, Jinan 250100, P. R. China

Tel./Fax: +86-531-88392983

E-mail address: haiyanhup@126.com.

Supplementary Figures

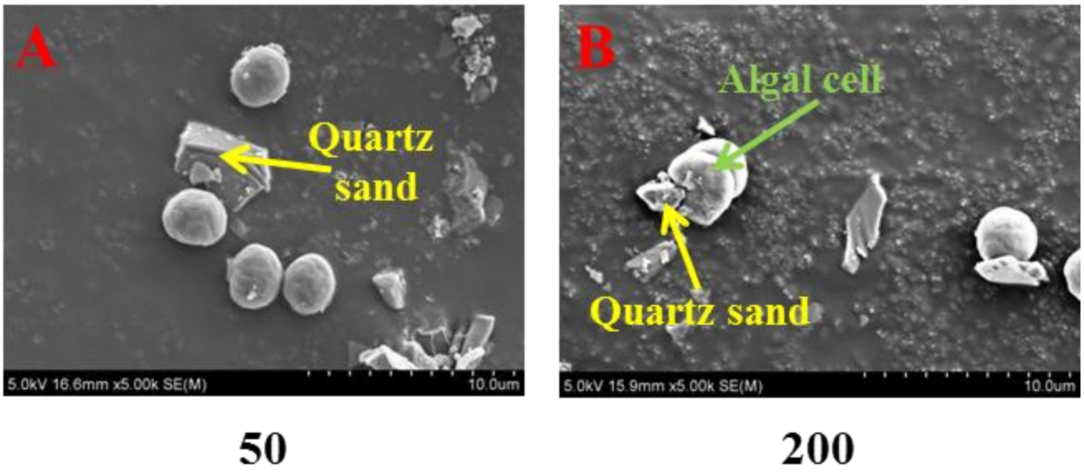

Fig. S1. SEM micrographs showing the effect of quartz sand on the integrity of *M. aeruginosa* cells during the coagulation process. (A: 50 mg/L quartz sand. B: 200 mg/L quartz sand.)

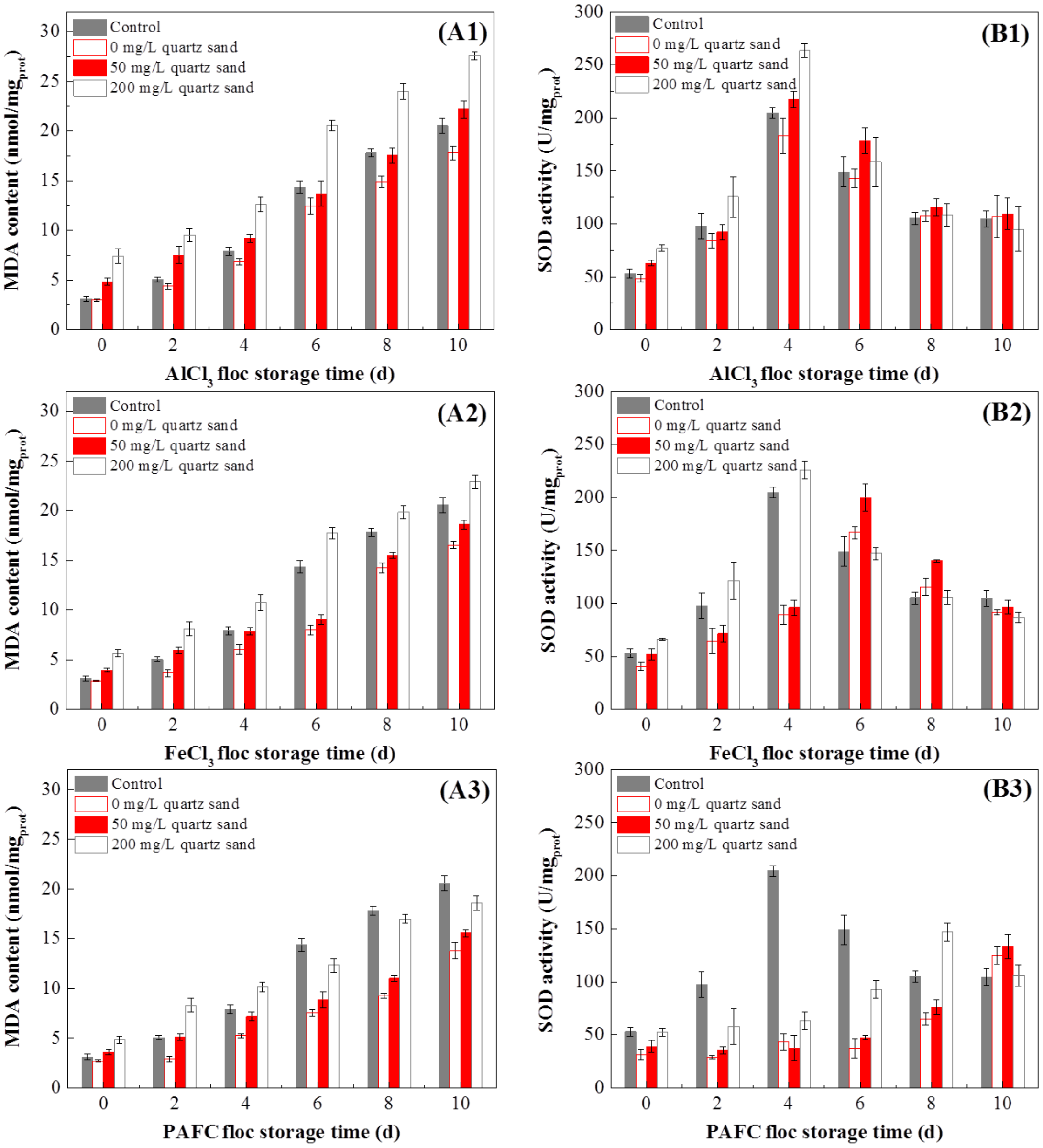

Fig. S2. The MDA content (A) and SOD activity (B) of $AlCl_3$ (A1, B1), $FeCl_3$ (A2, B2) and PAFC (A3, B3) flocs with 0, 50 and 200 mg/L quartz sand during 10 days of storage
